# Vaccination of the Eye-Lid

**Published:** 1882-12

**Authors:** A. W. Calhoun

**Affiliations:** Atlanta, Ga., Professor of Eye, Ear and Throat Diseases in Atlanta Medical College


### VACCINATION OF THE EYE-LID.

BY A. W. CALHOUN, M.D., ATLANTA, GA.,
Professor of Eye, Ear and Throat Diseases in Atlanta Medical College.

A certain author once wrote: "Happily, since the introduction of vaccination, the variolous ophthalmia is far less dangerous than formerly, when it led but too frequently to complete destruction of the sight." During the recent prevalence of small-pox, a case of accidental vaccination of the eye-lid, in a child, came under my observation and treatment, which, in point of violence of inflammation and destruction of the tissues involved, would equal the small-pox pustule, the bad results of which the above mentioned author so graphically describes.

The father of the child is a prominent and intelligent physician, doing a large practice in Alabama, and the following history, written by himself, will better describe the case, from the beginning of the disease up to the time it came into my keeping, than I could myself. I should state that the patient is a healthy, robust three-year-old boy. The letter is as follows:

"Early in the month of May, I vaccinated his little sister with a scab from a healthy child. I did not vaccinate him, as he was delicate, and there was no pressing necessity of doing so. About the 20th of the month, what was supposed to be a pimple was observed on the lower lid of the little fellow's left eye, and there was some photophobia and weeping of the eye. The swelling and other symptoms increased rapidly, but, being very busy at the time, I did not give him much attention, thinking it was simply a stye, with more local irritation than usual in consequence of his delicate health. The following Sunday, however, the infiammatory action became so violent that I was very apprehensive, and instinctively concluded that he must have been inoculated with lymph from his sister's arm. During the day he suffered a good deal and had a pretty high fever. Towards night all symptoms increased, and it was necessary to administer opiates to allay his suffering, and also ordered the application of cold compresses. It was impossible to separate the lids, and I felt very wretched, not knowing whether I had to deal with a vaccinated eye or a case of purulent ophthalmia. When he was undressed for bed, his grandmother discovered a 'sore' on his left breast, and called my attention to it. This I found to be a fully developed vaccine pustule, and, of course, cleared up all doubts as to the eye trouble. I immediately telegraphed you, and sought advice as to how the case should be treated. Never having come across such a case before in my experience or reading, I thought it possible that an effort might be made to abort the pustule, and thereby limit its destructive tendency. The next night you saw him, and gave us the benefit of your great kindness and skill.

"Now, an interesting question arises: How did he come in contact with vaccine virus? Certainly, there was none about the house within his reach, and he must have been inoculated by the nurse using the same towel for his face and his sister's arm, or lymph may have exuded from her arm on the pillow and in that way have come in contact with his eye, or else he may have touched the pustule on her arm and then scratched his eye. The nurse stoutly denies that she used the same towel for both children.

"The eye at the present time is perfectly clear and strong, but the lids are still somewhat red and infiltrated. The lower lid is intact, with the exception of the loss of half the cilia. The upper lid is lacerated, and has suffered loss of tissue, besides the destruction of half of the cilia. There will certainly be some permanent deformity, but time, I am confident, will rectify it to a considerable extent."

The child was seen immediately upon his arrival, and found in great pain—almost in convulsions. On the margin of the left upper and lower lids, near the inner canthus, and extending to the mucous lining of the lids, was a well-developed vaccine pustule; a second one on the left breast, a third just under the left ear, and a fourth on the edge of the left nostril. The lids and all the surrounding parts were intensely swollen, and as hard and immovable as it is possible for a lid to become. The secretions were of a muco-purulent character, but not very profuse. Appreciating the danger of such great pressure upon the cornea, an effort was made to open the lids in order to examine the ball, but the swelling made it impossible.

Opiates were given to the extent of relieving pain, the pustules were pricked and emptied, the lids were freely oiled and cold compresses applied. The general health was kept up by tonics, and the bowels attended to. This treatment was continued till the swelling subsided, when we were rejoiced to find the cornea and other portions of the ball intact, so far as destruction of the tissues was concerned. But the lids did not escape so lightly. The hair bulbs were so extensively destroyed that the eyelashes will be a permanent loss for about one-half of the length of the lids. The upper lid suffered a considerable loss of substance, which will remain a deformity till remedied.

The child most probably got the virus upon his fingers while asleep, and, by scratching, inoculated himself upon the lids, nose, breast and ear.

*Small-pox* pustules upon the lid, or even upon the ball, were in former times common enough to constitute an additional terror to the disease, but I can find no record of *vaccination* of these parts, though it might readily occur in any case.

A record is made of this case not only because of its rarity, but to put physicians and parents upon their guard, lest a similar accident should happen in their own hands, with perhaps less favorable results.

